# The level of BMP4 signaling is critical for the regulation of distinct T-box gene expression domains and growth along the dorso-ventral axis of the optic cup

**DOI:** 10.1186/1471-213X-6-62

**Published:** 2006-12-15

**Authors:** Hourinaz Behesti, James KL Holt, Jane C Sowden

**Affiliations:** 1 Developmental Biology Unit, Institute of Child Health, University College London, 30 Guilford Street, London, WC1N 1EH, UK

## Abstract

**Background:**

Polarised gene expression is thought to lead to the graded distribution of signaling molecules providing a patterning mechanism across the embryonic eye. Bone morphogenetic protein 4 (*Bmp4*) is expressed in the dorsal optic vesicle as it transforms into the optic cup. *Bmp4 *deletions in human and mouse result in failure of eye development, but little attempt has been made to investigate mammalian targets of BMP4 signaling. In chick, retroviral gene overexpression studies indicate that *Bmp4 *activates the dorsally expressed *Tbx5 *gene, which represses ventrally expressed *cVax*. It is not known whether the *Tbx5 *related genes, *Tbx2 *and *Tbx3*, are BMP4 targets in the mammalian retina and whether BMP4 acts at a distance from its site of expression. Although it is established that *Drosophila Dpp *(homologue of vertebrate *Bmp4*) acts as a morphogen, there is little evidence that BMP4 gradients are interpreted to create domains of BMP4 target gene expression in the mouse.

**Results:**

Our data show that the level of BMP4 signaling is critical for the regulation of distinct *Tbx2*, *Tbx3*, *Tbx5 *and *Vax2 *gene expression domains along the dorso-ventral axis of the mouse optic cup. BMP4 signaling gradients were manipulated in whole mouse embryo cultures during optic cup development, by implantation of beads soaked in BMP4, or the BMP antagonist Noggin, to provide a local signaling source. *Tbx2*, *Tbx3 *and *Tbx5*, showed a differential response to alterations in the level of BMP4 along the entire dorso-ventral axis of the optic cup, suggesting that BMP4 acts across a distance. Increased levels of BMP4 caused expansion of *Tbx2 *and *Tbx3*, but not *Tbx5*, into the ventral retina and repression of the ventral marker *Vax2*. Conversely, Noggin abolished *Tbx5 *expression but only shifted *Tbx2 *expression dorsally. Increased levels of BMP4 signaling caused decreased proliferation, reduced retinal volume and altered the shape of the optic cup.

**Conclusion:**

Our findings suggest the existence of a dorsal-high, ventral-low BMP4 signaling gradient across which distinct domains of *Tbx2*, *Tbx3*, *Tbx5 *and *Vax2 *transcription factor gene expression are set up. Furthermore we show that the correct level of BMP4 signaling is critical for normal growth of the mammalian embryonic eye.

## Background

Eye development begins in the fourth week of life in a human embryo, and at embryonic day (E) 8.5 in the mouse embryo. Bilateral protrusions of the developing forebrain neuroepithelium extend towards the surface ectoderm to form the optic vesicles. Invagination of the optic vesicle together with the overlying surface ectoderm forms the bi-layered optic cup and lens vesicle respectively. The distal region of the optic vesicle gives rise to the inner layer of the optic cup, the presumptive neural retina (NR). The proximal region of the optic vesicle forms the optic stalk and the outer layer of the optic cup forms the retinal pigmented epithelium (RPE). The optic cup is initially incomplete along the ventral surface and closure of the optic fissure marks the completion of the eye globe [1]. By around 6 weeks of human development (E13.5 in mouse) these critical stages of early eye morphogenesis are accomplished [2]. If disrupted, congenital malformations of the eye, such as anophthalmia, microphthalmia, and coloboma occur. These clinical conditions are heterogeneous and complex, probably involving genetic and environmental factors, however, a number of causative single gene mutations have been identified in humans or in murine models [3]. Elucidation of the interactions between key genes may lead to the identification of common pathways that are critical for the formation and growth of the embryonic optic cup.

During the morphological transition of the optic vesicle into the optic cup, formation of the dorsal (superior) part of the optic cup precedes formation of the ventral (inferior) part and closure of the optic fissure. As the optic cup forms in the chick and the mouse embryos, the ventral retinal region has a higher rate of proliferation compared with the dorsal retinal region [4,5]. Recent cell lineage tracing experiments carried out in chick embryos, using replication defective retroviruses suggested that the dorso-ventral (D-V) axis of the neural retina is divided into multiple domains of restricted gene expression which exhibit features of lineage compartments, with clones of cells originating within one compartment rarely crossing to neighbouring compartments [6]. Thus, differential regulation of cell proliferation within domains of gene expression across the D-V axis may be a mechanism driving the formation of the globe-like structure of the optic cup. The molecular mechanisms that pattern and shape this early growth phase of eye development are not well understood.

Signaling centres are believed to define 'patterning axes' within developing tissues through the diffusion of signaling molecules/morphogens and/or propagation of secondary relay mechanisms [7-10]. Asymmetric distribution of several secreted signaling molecules including bone morphogenetic protein (BMP), sonic hedgehog (SHH) and retinoic acid (RA) are implicated in regulating the development of the vertebrate eye [11,12]. RA signaling is needed for normal eye morphogenesis in mouse [13,14] and alteration of levels of RA signaling causes deletion or duplication of the ventral zebrafish retina [15,16]. SHH overexpression in zebrafish causes optic stalk hypertrophy and mutation of the human SHH gene is associated with holoprosencephaly and coloboma [17-19].

While important regulators of ventral retinal development have been identified [11,20-23], there is less understanding of the requirements for dorsal regulators in patterning and development of the dorsal retina or how they may affect formation of the ventral eye. The studies reported here focus on the role of *Bmp4*, which is specifically expressed in the dorsal retina of several vertebrate species including the mouse [24-26].

BMPs direct D-V patterning in both invertebrate and vertebrate embryos and are thought of as morphogens acting throughout a field of cells in a graded fashion [27-29]. Decapentaplegic (Dpp), the BMP4 homologue in *Drosophila melanogaster *has been at the forefront of morphogen studies exploring how endogenous morphogen gradients are shaped and interpreted to create boundaries for BMP target gene expression [30-32]. In vertebrates, a common role for BMP4 in tissue morphogenesis is emerging, notably in the limb, the mandible, and the beak [33-37]. Variation in the pattern of *Bmp4 *expression was recently shown to affect the shape and size of closely related finch beaks originally described by Darwin; *Bmp4 *misexpression in chick embryos, causes morphological transformations paralleling the beak morphology of the large ground finch [36,38].

BMP action in the nervous system includes effects on neural induction, cell fate determination, apoptosis and proliferation [39]. In the chick retina, *Bmp4 *has been implicated in regulating programmed cell death in the dorsal optic cup [40] and in regulating topographic mapping of retinal ganglion cells [41]. Overexpression of the BMP antagonist, Noggin, in chick, induces microphthalmia and coloboma, together with other ventral abnormalities, suggesting that endogenous BMPs have significant effects on the development of ventral optic cup structures [42]. Experiments in which optic vesicle explant cultures from *Bmp4 *null mice were treated with BMP4-soaked beads have shown that BMP4 signaling is essential, but not sufficient, for lens formation [26]. *Bmp4 *null embryos die at midgestation and optic vesicle development is arrested, hampering direct analysis of the role of *Bmp4 *in optic cup development. In *Bmp4*^+/- ^mice, the optic cup forms, but mature eyes show anterior segment abnormalities as well as an increased incidence of microphthalmia [43,44]. Deletions of human chromosome 14q, including the *BMP4 *gene have been identified in patients with anophthalmia [45-48].

Candidate downstream targets of BMP4 signaling in the mouse retina include members of the evolutionarily conserved T-box gene family of transcription factors. In *Drosophila*, Dpp regulates *omb*, a T-box gene critical for development of the fly visual system [31,49]. We have previously characterised the expression of three *omb*-related genes of the Tbx2 subfamily, *Tbx2*, *Tbx3*, and *Tbx5 *to be dorsally restricted in the human and mouse optic cup [50]. These genes are closely related and likely evolved from an ancestral *Tbx2*/*omb *gene via an unequal recombination event, followed by gene duplication, prior to the divergence of the invertebrate and vertebrate lineages [51]. Several studies in other species also support the idea that Tbx2 subfamily genes are regulated by BMP4. *Bmp4 *overexpression by electroporation in chick and *Xenopus *embryos induces ectopic *Tbx5 *expression in the retina [41,52]. In the chick, *Tbx3 *expression in the limb is also dependent on BMP signaling [53,54]. There is not yet functional evidence for the importance of the Tbx2 subfamily genes in mammalian eye development, however misexpression of *Tbx5 *in chick embryos, perturbs retinotectal projections [41]. Recent work has also shown that *Tbx2b *(*tbx-c*) in zebrafish may be important specifically for differentiation of retinal neurons in the dorsal retina [55].

Here we have developed the use of whole mouse embryo culture to test the hypothesis that BMP4 signaling regulates T-box gene expression in the developing mouse eye. This is the first time that implantation of growth factor soaked beads have been used to examine patterning of the developing mouse eye *in vivo*. We examined whether perturbation of BMP4 levels affect optic cup growth and shape. We report that *Tbx2*, *Tbx3*, and *Tbx5 *are all responsive to manipulations of BMP4 signaling, but that each gene responds differently. We found that increasing BMP4 signaling by addition of exogenous BMP4 alters the molecular patterns of gene expression across the D-V axis in a ventral shift, while addition of the BMP antagonist, Noggin induces a dorsal shift. These changes in gene expression are associated with a reduced retinal volume, primarily due to reduced cell proliferation and alterations in the axial lengths of the optic cup, indicating that correct levels of BMP signaling are important for the growth and the shape of the embryonic eye.

## Results

### Nested expression of *Bmp4*, *Tbx2*, *Tbx3 *and *Tbx5 *in the mouse optic cup

**Figure 1:**
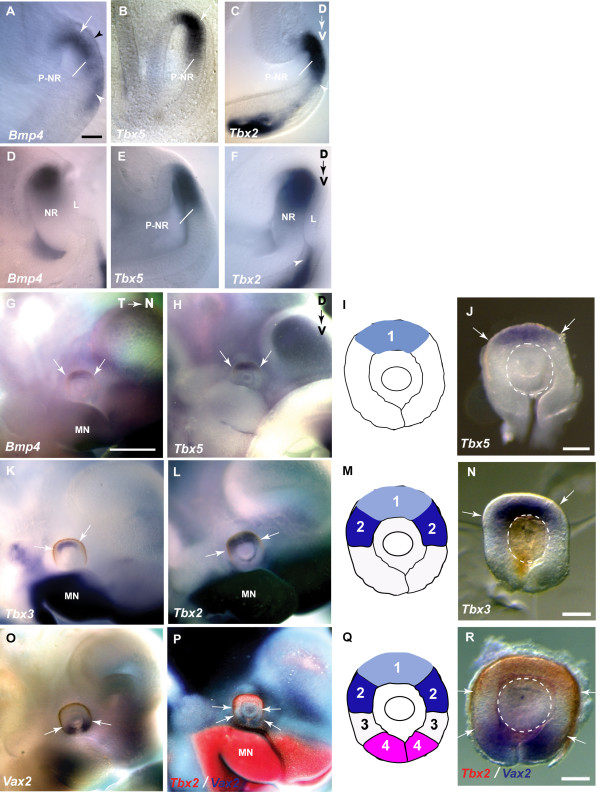
**Expression of *Bmp4*, *Tbx2, Tbx3*, *Tbx5*, and *Vax2 *during optic cup formation**. **(A-C) ***Bmp4 *co-expressed with *Tbx5 *and *Tbx2 *in the optic vesicle at E9.5 (arrows; 15 somite stage). Black arrowhead in A indicates *Bmp4 *expression in the surface ectoderm. White arrowheads indicate expression in mesenchyme ventral to the developing eye. **(D-F) ***Bmp4*, *Tbx5 *and *Tbx2 *expression in the invaginating optic vesicle at E10.5 (24–29 somite stage). **(G, H**) *Bmp4 *and *Tbx5 *expression at E11.5 in the optic cup in dorsal domain 1, shown schematically in **I**. **(J) ***Tbx5 *expression in dorsal domain 1 in an E11.5 dissected optic cup. (**K**) *Tbx3 *expression in dorsal domain 1 and its extension into dorsal domain 2 in the temporal neural retina. **(L) ***Tbx2 *expression in domains 1 and 2, shown schematically in **M**. **(N) ***Tbx3 *expression in an E11.5 dissected optic cup. **(O) ***Vax2 *expression at E11.5 in the ventral optic cup in domain 4. **(P) **Double labeling of *Tbx2 *(red) and *Vax2 *(blue) shows a domain in between domain 2 and domain 4, which does not express either marker, and is designated domain 3, shown schematically in **Q**. **(R) ***Tbx2 *(red) and *Vax2 *(blue) expression in an E11.5 dissected optic cup, revealing domain 3 in white. Arrows in G-R indicate the boundaries of gene expression domains. Dashed circles in J, N, R demarcate the lens vesicle. A-F are coronal sections. Scale bars: A-F, 0.05 mm; G, H, K, L, O, P, 0.5 mm; J, N, R, 0.1 mm. Abbreviations: L, lens placode; MN, mandibular process of the first branchial arch; NR, neural retina; P-NR, presumptive neural retina; D-V indicates the dorso-ventral axis, N-T indicates the naso-temporal axis.

The expression pattern of *Bmp4 *and members of the Tbx2 subfamily were compared during development of the optic cup in mouse embryos. *Bmp4 *expression was detected in the dorso-distal optic vesicle from E9.5 (Fig. 1A, somite stage 15) in the region of neuroepithelium contacting the surface ectoderm. The distal optic vesicle is the presumptive neural retina. *Tbx5 *and *Tbx2 *were expressed in a similar region of the presumptive neural retina as *Bmp4 *at this stage (Fig. 1B,C). *Bmp4 *and *Tbx2*, but not *Tbx5 *were expressed in the mesenchyme ventral to the optic vesicle (white arrowheads in Fig. 1A and 1C). *Bmp4 *was also expressed in the surface ectoderm (Fig. 1A, black arrowhead), as previously reported [26].

During optic cup invagination at E10.5, the *Bmp4 *expression domain in the dorsal neural retina remained similar to that of *Tbx5 *and *Tbx2 *(Fig. 1D,E,F). By E11.5, *Tbx5 *and *Bmp4 *were co-expressed in the dorsal-most region of the optic cup, designated domain 1 (Fig. 1G–J), whereas *Tbx2 *expression in the dorsal retina was broader and extended ventrally beyond domain 1 into a second domain designated domain 2 (Fig. 1L,M). A third Tbx2 subfamily gene, *Tbx3*, was also expressed in domain 1 and extended into domain 2 in the temporal, but not nasal, neural retina (Fig. 1K,N). *Tbx2, Tbx3 *and *Bmp4 *were also expressed in the mandibular and maxillary processes, ventral to the optic cup (Fig. 1G,K,L). Expression domains 1 and 2, were compared with the expression domain of a ventral marker, the *Vax2 *homeobox gene. *Vax2 *is expressed in the ventral-most region, designated domain 4 (Fig. 1O). The region lying between domains 2 and 4, which expresses neither *Tbx2*, nor *Vax2*, was designated domain 3 (Fig. 1P–R).

*Bmp4 *is thus highly expressed within the dorso-distal eye primordia, providing a dorsal source of BMP4 signaling throughout formation of the optic cup. The spatio-temporal overlap in the expression patterns of *Bmp4*, *Tbx2, Tbx3 *and *Tbx5 *in the dorsal eye supports the idea that BMP4 is an upstream regulator of these Tbx2 subfamily genes during mouse eye development. That *Tbx2 *and *Tbx3 *are expressed in a broader domain than *Bmp4*, suggests that these genes may respond to BMP4 signaling which is acting some distance from the source of *Bmp4 *expression. To test the hypothesis that *Bmp4 *expressed in domain 1, regulates T-box gene expression in domains 1 and 2, we aimed to increase the level of BMP4 signaling by implanting growth factor soaked agarose beads into the mouse eye primordia and then observed the effect on gene expression domains.

**Figure 2:**
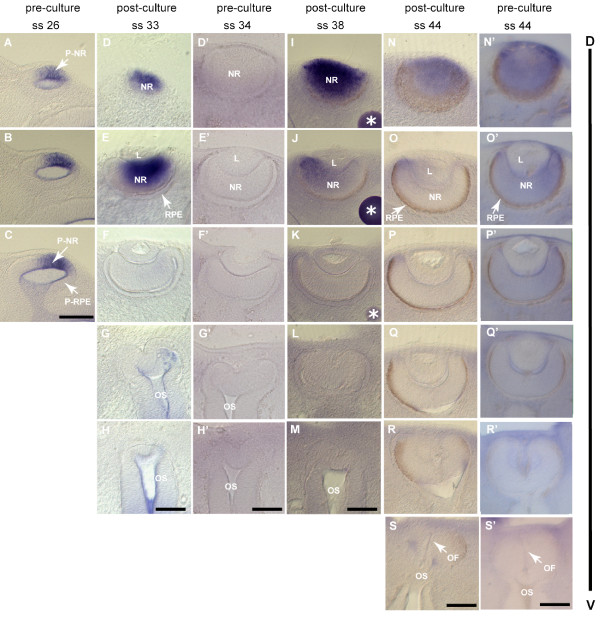
**Optic cup development in whole mouse embryo culture**. A-S' are serial transverse sections through the developing optic vesicle and optic cup, ordered with dorsal (D) sections uppermost. A-H, I-M, and N-S show *Tbx5 *expression (in blue). **(A-C) **E10.5 optic vesicle (not cultured). **(D-H) **Post-culture early optic cup. Compare with A-C for pre-culture stage of development and to **D'-H' **for a stage matched non-cultured optic cup. Invagination to form an optic cup with a thick neural retina (NR) surrounded by a thin layer of presumptive retinal pigmented epithelium (RPE), and formation of a lens vesicle (L) occur normally during culture. **(I-M) **Post-culture optic cup implanted with a control bead, showing normal eye morphology and gene expression. (**N-S**) Post-culture late optic cup. Compare with D'-H' for the approximate pre-culture stage of development and with **N'-S' **for the non-cultured stage matched equivalent. Growth of the NR, pigmentation of the RPE, and apposition of the optic fissure walls (indicated by arrows in S and S') proceed normally during culture. Scale bars: 0.1 mm. Abbreviations: D, dorsal; L, lens vesicle; NR, neural retina; P-NR; presumptive neural retina; OF, optic fissure; OS, optic stalk; RPE, retinal pigmented epithelium; P-RPE, presumptive RPE; ss, somite stage; V, ventral.

We developed a system using whole mouse embryo culture, analogous to the method of bead implantation used in the developing chick embryo *in ovo *[56,57]. In contrast to widespread retroviral or plasmid overexpression approaches, growth factor soaked beads allow the establishment of a local signaling source to test the effect of diffusion of signaling factors across a field of cells. Mouse embryos were cultured using established conditions [58]. Control experiments were performed to ensure, first, that normal optic cup morphogenesis was achieved, and second, that implantation of beads soaked in the biologically inactive protein, bovine serum albumin (BSA), did not disrupt normal morphogenesis or gene expression. We found that normal eye development occurred *ex utero *during whole mouse embryo culture; embryos were cultured from three time points: E9.5 (optic vesicle); E10.5 (optic vesicle to optic cup transition); and E11.5 (early optic cup). In CBA/Ca embryos, optic vesicle invagination initiates at 24–26 somites and the optic cup forms by late E10.5, at 33–35 somites. This process occurred faithfully in culture (Fig. 2A–C, pre-culture optic vesicle, Fig. 2D–H post-culture optic cup compare with Fig. 2D'–H' stage matched non-cultured optic cup). Normal growth and pigmentation of the optic cup also occurred in culture (Fig. 2N–S post-culture optic cup compare to Fig. 2N'–S' stage matched non-cultured optic cup) and normal patterns of T-box gene expression were observed in post-culture embryos (Fig. 2 and data not shown). Implantation of BSA-soaked agarose beads around the optic vesicle and within the lens of the optic cup did not disrupt morphogenesis or gene expression (Fig. 2I–M and data not shown). Hence, embryo culture, combined with bead implantation provides a useful *ex utero *model for investigation of how alteration of BMP signaling affects patterning of the mammalian optic cup.

### Exogenous BMP4 induces T-box gene expression in the developing optic cup

BMP4-soaked beads were implanted in the dorso-temporal or dorso-nasal peri-ocular mesenchyme adjacent to the optic vesicle of E9.5 and E10.5 embryos, which were cultured overnight. Beads soaked in recombinant BMP4 protein have previously been shown to be functionally active in the mouse embryo [26]. The bead implantation site was selected to provide an ectopic source of BMP4 signaling, and the effect of growth factor perturbation in the operated eye was compared to the contralateral non-treated eye in each embryo.

**Figure 3:**
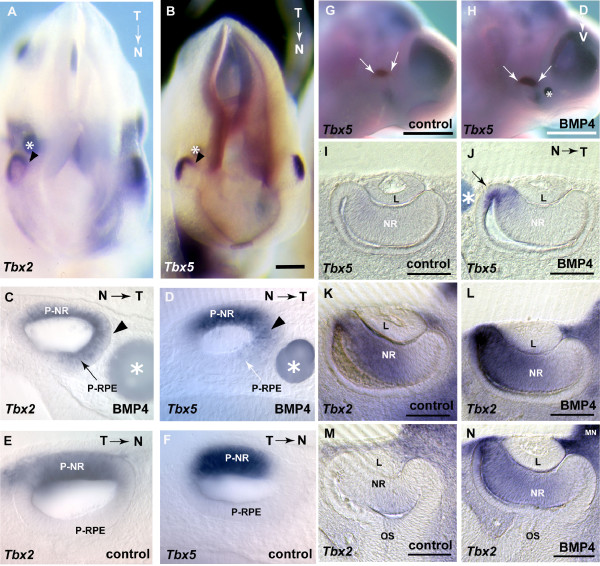
**Induction of *Tbx2 *and *Tbx5 *expression by exogenous BMP4. (A, B) **Dorsal views of post-culture embryos showing ectopic expression of *Tbx2 *(A) and *Tbx5 *(B) in the proximal optic vesicle (arrowheads) after implantation of a BMP4-soaked bead in the peri-ocular mesenchyme (asterisks). Expression is extended into the presumptive RPE on the treated side but restricted to the presumptive neural retina on the contralateral non-treated side shown by transverse sections through the optic vesicle **(**arrowheads,**C-F)**. **(G, H) **Lateral views of a post-culture embryo showing normal *Tbx5 *expression in the non-treated eye (G) and increased *Tbx5 *expression after implantation of a BMP4-soaked bead in the periocular mesenchyme (H). Arrows demarcate the *Tbx5 *expression domain. **(I-J) **Transverse sections of the non-treated and BMP4-treated contralateral eyes showing the extended *Tbx5 *expression into the presumptive RPE next to the BMP4-soaked bead (arrow). **(K-N) **Serial transverse sections showing normal *Tbx2 *expression in a control eye (K, M) and the extension of *Tbx2 *expression ventrally after implantation of a BMP4-soaked bead in the treated eye (L, N). Sections M and N are immediately ventral to sections K and L. Scale bars: A, B, 0.1 mm; C-F, 0.05 mm; G, H, 0.5 mm; I-N, 0.1 mm. Abbreviations: L, lens vesicle; MN, mandibular region of the first branchial arch; MX, maxillary region of the first branchial arch; N, nasal; NR, neural retina; P-NR, presumptive neural retina; OS, optic stalk; P-RPE, presumptive retinal pigmented epithelium; T, temporal.

Exogenous BMP4 induced ectopic *Tbx2 *and *Tbx5 *expression in the proximal optic vesicle, the presumptive RPE (Table 1; Fig. 3A–F). The extension of the normal expression domain is visible in dorsal views of the embryos (Fig. 3A,B). Transverse sections showed *Tbx2 *and *Tbx5 *up-regulation in the presumptive RPE that normally does not express the T-box genes, with *Tbx2 *showing the most marked response (Fig. 3C,D). On the non-treated side, as well as in control embryos implanted with BSA-soaked beads, T-box gene expression was restricted to the dorso-distal optic vesicle (Fig. 3A,B,E,F and data not shown).

**Table 1:** Changes in gene expression by BMP4, Noggin, and BSA-soaked beads at E9.5-E11.5

**Protein**	**Stage**	**Gene**	**Gene expression altered**
BMP4	E11.5 (bead in lens)	*Tbx5*	10/12 (+)
		*Tbx2*	11/13 (+)
		*Tbx3*	3/3 (+)
		*Vax2*	4/4 (-)
	E10.5 (bead in mes)	*Tbx5*	3/8 (+)
		*Tbx2*	1/4 (+)
		*Tbx3*	0/2
		*Vax2*	0/2
	E9.5 (bead in mes)	*Tbx5*	3/3 (+)
		*Tbx2*	2/2 (+)

BSA	E11.5 (bead in lens)	*Tbx5*	0/5
		*Tbx2*	0/6
		*Tbx3*	0/1
		*Vax2*	0/6
	E10.5 (bead in mes)	*Tbx5*	0/3
		*Tbx2*	0/2
	E9.5 (bead in mes)	*Tbx5*	0/2

Noggin	E10.5 (bead in mes)	*Tbx5*	6/6 (-)
		*Tbx2*	4/4 (-)
		*Tbx3*	0/2
		*Vax2*	0/3

Mes, mesenchyme; Altered gene expression after culture with bead: + indicates induction; - indicates repression. Bead implantation was into nasal peri-ocular mesenchyme.

To assess the effect of changing the level of BMP4 signaling during optic cup formation, BMP4-soaked beads were implanted adjacent to the optic vesicle of early E10.5 (22–26 somites) embryos, prior to optic vesicle invagination, and embryos were cultured through the invagination process. *Tbx5 *expression was never extended to the ventral retina and remained dorsally restricted (Fig. 3G,H). Sections through the dorsal eye showed that *Tbx5 *was induced in the presumptive RPE adjacent to the bead, consistent with observations at E9.5 (Fig. 3I,J). Rarely, exogenous BMP4 extended *Tbx2 *expression into domain 3 in the ventral retina (Table 1; Fig. 3K–N). No change in expression of *Vax2 *or *Tbx3 *was observed after BMP4 bead implantation (Table 1). BMP4-soaked beads placed temporal to the eye gave similar results to nasal implantation (nasal data are shown in Table 1).

*Tbx2 *and *Tbx5 *could not be induced in the ventral-most region, domain 4, even when BMP4-soaked beads were placed ventral to the optic vesicle in E9.5 and E10.5 culture (data not shown). Exogenous BMP4 did not disrupt the process of optic vesicle invagination; normal optic cup formation occurred during the culture period. In summary, exogenous BMP4 protein induced ectopic expression of both *Tbx2 *and *Tbx5 *during optic cup formation.

### Noggin abolishes *Tbx5 *and reduces *Tbx2 *expression

**Figure 4:**
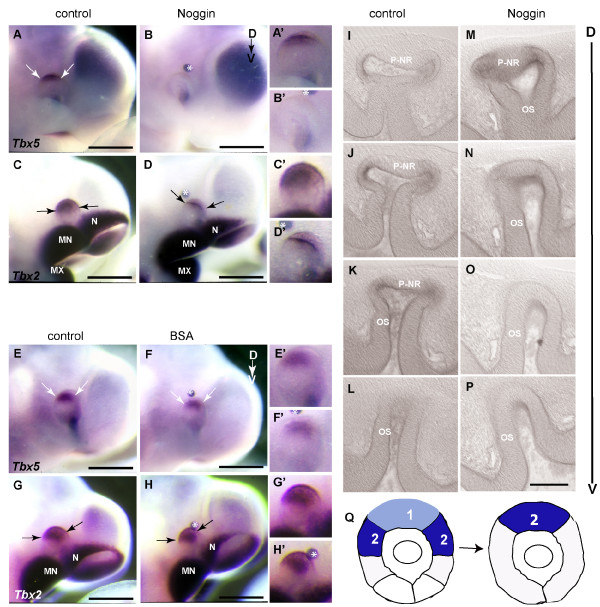
**Repression of *Tbx2 *and *Tbx5 *expression by exogenous Noggin. (A) **Post-culture embryo showing *Tbx5 *expression in the non-treated dorsal optic cup. **(B) **Contralateral Noggin-treated eye, showing absence of *Tbx5 *expression in the optic cup. **(C) **Post-culture embryo showing *Tbx2 *expression in the non-treated dorsal optic cup. **(D) **Contralateral Noggin-treated eye showing a reduced *Tbx2 *expression domain. *Tbx5 *expression **(E, F) **and *Tbx2 *expression **(G, H) **are not altered in non-operated and BSA-treated eyes respectively of post-culture embryos. A'-H' show higher magnifications of eyes in A-H. Arrows indicate boundaries of gene expression domains. Implanted beads are marked by asterisks. **(I-P) **Serial transverse vibratome sections through the optic cup showing altered morphology after Noggin treatment, with the dorsal-most sections in upper panels. (I-L) Post-culture non-treated eye showing invagination of the presumptive neural retina (P-NR) in the ventral optic cup. (M-P) Contralateral Noggin-treated eye showing dorsal extension of the optic stalk region as compared to the non-treated eye. **(Q) **Schematic representation of normal *Tbx2 *and *Tbx5 *expression domains in a lateral view of the optic cup, and the dorsal shift of the expression domains induced by exogenous Noggin. Scale bars: A-H, 0.5 mm; I-P, 0.1 mm. Abbreviations; MN, mandibular process of the first branchial arch; MX, maxillary process of the first branchial arch; N, nasal process; P-NR, presumptive neural retina; OS, optic stalk.

To provide further evidence supporting the role of BMP4 signaling in regulating T-box gene expression, we used the BMP antagonist, Noggin, to block BMP4 signaling in the eye (Fig. 4). Noggin has high affinity for BMP4 and prevents BMP receptor-ligand association, hence disrupting the signaling pathway [59].

Noggin-soaked beads were implanted close to the site of endogenous *Bmp4 *expression, dorsal to the optic vesicle of E10.5 embryos. Noggin abolished *Tbx5 *expression (Fig. 4A,B,A',B'), but only reduced the *Tbx2 *expression domain to the dorsal-most region of the eye, domain 1, and did not markedly affect *Tbx3 *expression (Fig. 4C,D,C',D' and [see Additional File 1A, B]; Table 1). Implantation of BSA-soaked beads in the same position as the Noggin-soaked beads had no effect on either *Tbx5 *or *Tbx2 *expression (Fig. 4E–H,E'–H'). Analysis of *Vax2 *after Noggin treatment showed that *Vax2 *expression was maintained ([see Additional File 1C, D]; Table 1).

Analysis of the morphology of manipulated eyes revealed that dorsal optic vesicle invagination initiated in the presence of Noggin. However, the ventral region of the operated eye, showed a marked delay in the invagination process (Fig. 4M–P) when compared to the contralateral control eye (Fig. 4I–L; n = 6/8). In operated eyes, invagination to form the optic cup had not occurred in ventral sections, and only the single layered neuroepithelium of the optic vesicle was observed (Fig. 4N–P), By contrast, formation of the bi-layered optic cup and increased growth of the presumptive neural retina was advanced in the ventral region of control eyes (Fig. 4J–L).

In summary, at E10.5, Noggin-soaked beads induced a dorsal shift in expression domains by repressing expression of *Tbx5 *in domain 1 and *Tbx2 *in domain 2. Thus, the dorsal-most region of the retina acquired the expression profile of domain 2, in that it was *Tbx2*-positive, *Tbx5*-negative (Fig. 4Q). Although *Tbx2 *and *Tbx5 *expression was altered by increased or decreased levels of BMP4 signaling *, Tbx3 *and *Vax2 *expression appeared tolerant of the same changes in BMP signaling levels.

### Effect of disruption of BMP4 signaling on dorso-ventral patterning

To explore whether the concentration of BMP4 is important for defining distinct domains of gene expression in the mouse optic cup, and whether genes show different responses to alterations in the level of signaling, BMP4-soaked beads were placed centrally in the eye, within the lens vesicle of optic cup stage E11.5 embryos. Exogenous BMP4 added to the centre of the optic cup is at an equivalent distance from both the dorsal and the ventral retina, and by raising BMP4 levels throughout the eye, may disrupt the normal putative BMP4 (dorsal-high, ventral-low) signaling gradient.

**Figure 5:**
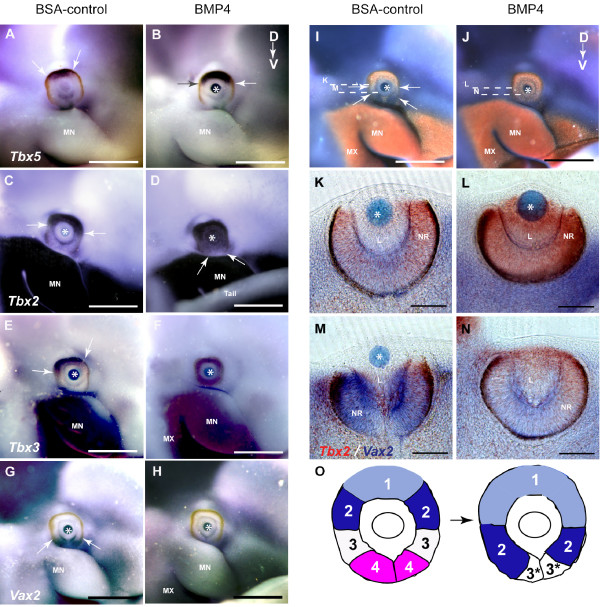
**Ventral shift of gene expression along the D-V axis of the optic cup after BMP4 treatment**. Beads are indicated with asterisks and domains of gene expression are demarcated with arrows. **(A) **Post-culture embryo showing normal dorsal *Tbx5 *expression in the control optic cup. **(B) **Contralateral BMP4-treated eye, showing extension of the *Tbx5 *expression domain. **(C) **Post-culture embryo showing normal dorsal *Tbx2 *expression in the control BSA-treated optic cup. **(D) **Contralateral BMP4-treated eye, showing extension of the *Tbx2 *expression domain into the ventral optic cup and induction of expression in the lens. **(E) **Post-culture embryo showing normal dorsal *Tbx3 *expression in the control BSA-treated optic cup. **(F) **Contralateral BMP4-treated eye, showing induction of *Tbx3 *expression throughout the ventral optic cup. **(G) **Post-culture embryo showing normal ventral expression of *Vax2 *in the control BSA-treated optic cup. **(H) **Contralateral BMP4-treated eye, showing complete absence of *Vax2 *expression. **(I, J) **Double *in situ *hybridisation of *Tbx2 *expression (red) and *Vax2 *expression (blue) in control BSA-treated (I) and contralateral BMP4-treated eyes (J). **(K-N) **Transverse sections through the planes indicated in I and J, showing induction of ectopic *Tbx2 *expression (K, L) and repression of *Vax2 *expression (M, N) upon BMP4 treatment. **(O) **Schematic representation of gene expression domains 1–4 along the D-V axis of the whole mount optic cup and the ventral shift of domains upon BMP4 treatment. Domain 3* is *Tbx2*-negative, *Tbx3*-positive, *Vax2*-negative. Scale bars: A-J, 0.5 mm; K-N, 0.1 mm. Abbreviations: L, lens vesicle; MN, mandibular process of the first branchial arch; MX, maxillary process of the first branchial arch; NR, neural retina.

Exogenous BMP4 resulted in the extension of *Tbx5 *expression from domain 1 across the dorsal half of the retina, covering the normal site of *Tbx2 *expression in domain 2 (Table 1; Fig. 5A,B). Ectopic *Tbx5 *expression never crossed to the ventral half of the retina. *Tbx2 *was expanded into the ventral retina to cover domain 3 (Fig. 5C,D), and *Tbx3 *was expanded across domains 2, 3 and 4 (Fig. 5E,F). Strikingly,*Vax2 *expression in domain 4, was completely abolished in BMP4-treated eyes (Fig. 5G,H). *Tbx2*, but not *Tbx3 *or *Tbx5*, was also induced in the lens (which does not normally express *Bmp4*; Fig. 5C,D). Double *in situ *hybridisation for *Tbx2 *and *Vax2 *confirmed the expansion of *Tbx2 *to entirely cover domain 3, but not 4, and the loss of *Vax2 *in domain 4 (Fig. 5I–N). Control experiments, carried out by implantation of BSA-soaked beads in the same location as BMP4-soaked beads either in the contralateral eye of the same embryo, or in parallel on somite-matched embryos never showed alterations in patterns of gene expression (Fig. 5C,E,G,I; Table 1, and data not shown).

Our findings show that exogenous BMP4 provided to the central region of the eye has shifted the normal gene expression boundaries ventrally by one or more domains (Fig. 5O), suggesting that the molecular division of the retina into gene expression domains is dependent on precise levels of BMP4 signaling. Although all genes showed a ventral shift, their response was non-uniform suggesting that additional regulatory mechanisms exist. Domain 1 (*Tbx5*-positive, *Tbx2*-positive), was expanded ventrally to cover the former site of the *Tbx5*-negative, *Tbx2*-positive domain 2; whereas the latter domain is now shifted to the more ventral former site of domain 3. *Tbx3 *showed a different response and shifted ventrally by two domains into the entire ventral retina. Domain 3*, which shows the expression profile *Tbx5*-negative, *Tbx2*-negative, *Tbx3*-positive is in the ventral-most region of BMP4-treated eyes and the *Vax2*-positive domain 4 is lost in this ventral shifting of expression domains.

To assess BMP signaling in the optic cup we performed immunohistochemistry for the Phospho-Smad 1/5/8 proteins which transduce the extracellular BMP signal to the nucleus [60]. Phospho-Smad 1/5/8 labeling was highest in the dorsal retina (consistent with a previous report [61]), and in the lens in non-treated control eyes, while labeling was detected across a wider region in the retina of BMP4-treated eyes [see Additional File 1F–I]. These data suggest that BMP signaling in the retina extends beyond the *Bmp4 *expression domain upon addition of exogenous BMP4 [see Additional File 1E–I]. BMP4 has previously been shown to upregulate the *Msx2 *homeobox gene in other systems [62-64]. Since *Msx2 *is expressed in the mouse optic cup [65] we tested the effect of BMP4 bead implantation and compared the *Msx2 *response to that of the Tbx2 subfamily genes. Exogenous BMP4 induced *Msx2 *gene expression in a different pattern to any of the other marker genes tested; high level ectopic *Msx2 *expression was detected in the lens, optic cup and surface ectoderm, as compared with control eyes [see Additional File 1J, K].

### Change in eye size and shape after BMP4 treatment

**Figure 6:**
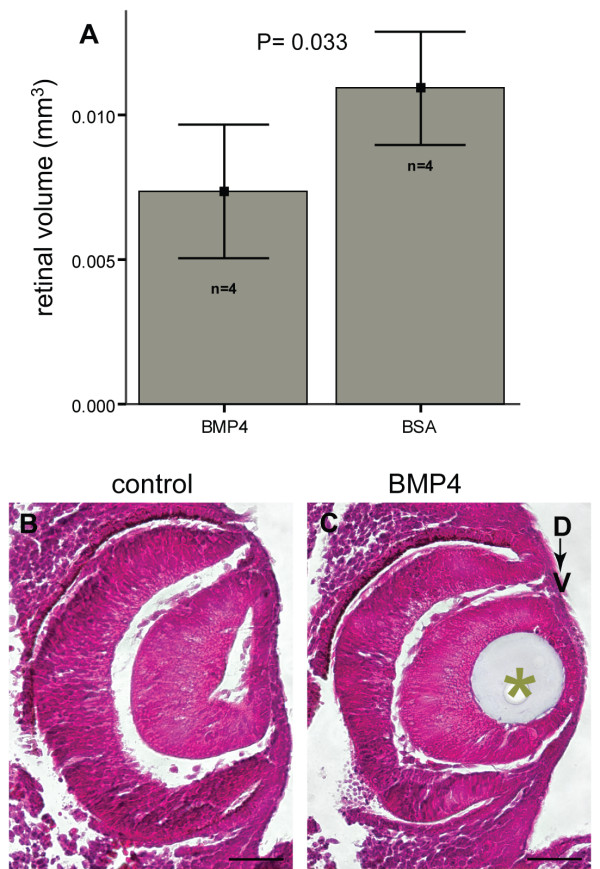
**Effect of BMP4 treatment on eye size and shape. (A) **Retinal volume in BMP4-treated and contralateral BSA-treated eyes estimated from four post-culture embryos and represented by bar charts as mean ± 1 S.D. p = 0.033 by the paired t-test indicates a significant reduction in retinal volume in BMP4-treated eyes. **(B, C) **Coronal sections of control (B) and contralateral BMP4-treated (C) eyes stained with H&E and showing a reduction in eye size and retinal thickness upon BMP4-treatment. Scale bars: 0.05 mm.

BMP4-treated eyes of E11.5 embryos, displaying a ventral shift in Tbx2 subfamily gene expression domains, were examined for morphological abnormalities. These eyes appeared smaller in size and this difference was analysed by estimating retinal volumes. Retina from BMP4-treated eyes were consistently smaller than those of contralateral eyes treated with BSA control beads (n = 4 embryos; mean volume ± 1 S.D., BMP4-treated retina = 7.35 × 10^-3 ^mm^3 ^± 2.3 × 10^-3^; mean volume ± 1 S.D., BSA-treated retina = 10.90 × 10^-3 ^mm^3 ^± 1.95 × 10^-3^; p = 0.033; Fig. 6A).

We next investigated how the eye had changed in shape, and whether there was a change in the axial lengths of the optic cup associated with the changed gene expression patterns. For each embryo, the D-V, naso-temporal (N-T) and proximo-distal (P-Di) axes of the BMP4-treated eye were measured and compared to the control eye of the same embryo. The percentage change in axial length between treated and contralateral control eyes were calculated (Table 2). In the BMP4-treated group, the D-V axis of the BMP4-treated eye was smaller than the non-treated contralateral eye in every case (n = 25, P < 0.0001). By contrast there was no significant difference between BSA-treated eyes and the non-treated control (n = 8; p = 0.07). Neither was there a significant natural variance between the axial length of right and left eyes of non-treated cultured embryos (n = 10; p = 0.10).

**Table 2:** Changes in axial length of the optic cup after BMP4 treatment

	**Treatment Group**	**n.**	**Mean percentage difference ± 1.S.D.**	**P-value**
**D-V axial length**	none	10	98.1% ± 3.50	0.11
	BMP4	25	92.33% ± 3.83	<0.0001
	BSA	8	95.26% ± 7.77	0.07

**N-T axial length**	none	10	99.97% ± 2.92	1
	BMP4	25	96.66% ± 4.93	0.001
	BSA	8	97.87% ± 3.50	0.15

**P-Di axial length**	BMP4/BSA	4	93.8% ± 6.9	0.58

100% indicates no change; D-V, dorso-ventral; N-T, naso-temporal; P-Di, proximo-distal

Similarly, the N-T axis of BMP4-treated eyes was reduced, although the reduction along the N-T axis of BMP4-treated eyes was less pronounced than that along the D-V axis (Table 2, n = 25; p = 0.001). There was no significant difference in the BSA-treated group (n = 8, p = 0.15), nor in non-treated cultured embryos (n = 10, p = 1). By contrast, the P-Di axis (from the ventricular edge of the retina to the surface ectoderm) was unchanged by the exogenous BMP4 (n = 4, 0.58).

These data show that the addition of exogenous BMP4 reduced optic cup size and the D-V and N-T axial lengths, though not the P-Di axial length, thus inducing a change in the size and the shape of the optic cup. Histological sections also showed the small eye size and the abnormally thin neural retina of BMP4-treated optic cups (Fig. 6B,C).

### Cell proliferation and cell death in BMP4-treated eyes

**Figure 7:**
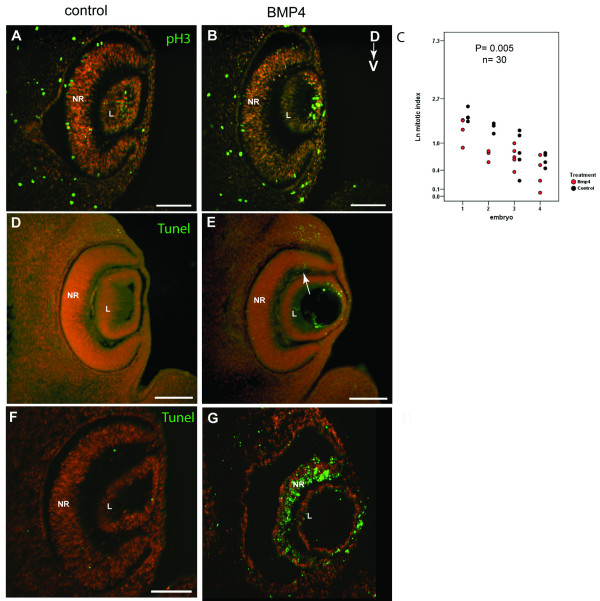
**Effect of BMP4 treatment on proliferation and cell death**. **(A, B) **pH3-positive cells (green) in retinal sections of control and BMP4-treated contralateral eyes of a post-culture embryo. Sections are counterstained with propidium iodide (red). **(C) **Graph showing the mitotic index per section per eye in BMP4-treated optic cups, compared with contralateral control optic cups (n = 30 sections from 4 embryos; p = 0.005 by ANCOVA). Data was Ln transformed for normalisation. Between embryo variability likely reflects the differences in growth rate of individual embryos. **(D, E) **Increased dorsal apoptosis detected by the TUNEL assay (green) in BMP4-treated eyes (arrow in E) compared to contralateral control eyes (D) of post-culture embryos. **(F, G) **Apoptosis detected in eyes of post-culture embryos after high dose BMP4 treatment. Widespread apoptosis detected throughout the neural retina. Scale bars: 0.1 mm. Abbreviations: D, dorsal; L, lens vesicle; NR, neural retina; pH3, phospho-histone H3; TUNEL, Terminal deoxynucleotidyl transferase-mediated dUTP Nick End Labeling; V, ventral.

The differences in gene expression domains and optic cup size/shape observed after the addition of exogenous BMP4 to developing optic cups could be due to regional decreases in retinal cell proliferation or increases in apoptosis (or both). To establish whether the altered gene expression domains correlated with regionalised changes in cell proliferation, retinal sections were immuno-labelled using the pH3 antibody, which labels mitotic cells in the retina at the ventricular surface. BMP4-treated embryos (n = 4) were examined for differences in the number of pH3-positive cells either throughout the retina, or in the dorsal region (containing domains 1 to 3) and the ventral region (containing domain 4). There was a significant overall decrease in the mitotic index (number of pH3-labelled cells as a percentage of total cells) in BMP4-treated compared with contralateral non-treated eyes in cultured embryos (p = 0.005, 2-way ANCOVA, n = 30 sections; Fig. 7A–C). Comparisons of both dorsal and ventral cell counts between eyes showed that the number of pH3-labelled cells were significantly reduced after BMP4 treatment in both the dorsal (p = 0.007; 2-way ANOVA, n = 44 sections) and the ventral (p = 0.018; 2-way ANOVA, n = 44 sections) retina [see Additional File 2A, B]. Non-treated eyes showed a significantly higher level of mitosis (pH3 per mm^2^) in the ventral retina, compared to the dorsal region (p = 0.013; 2-way ANOVA, n = 19 sections, data not shown), but this difference was abolished in BMP4-treated eyes (p = 0.4, data not shown).

Finally we used TUNEL labeling to assess whether the altered gene expression domains observed after BMP4 treatment were associated with increased or altered patterns of apoptosis. Regionalised apoptosis could cause tissue ablation and loss of gene expression domains. For example ablation of ventral tissue could cause loss of the *Vax4 *expression domain and reduction of eye size. BMP4 has been shown to promote apoptosis in other systems [57,62,66,67].

In control eyes, TUNEL labeling was detected in the region of the optic disc and the optic stalk. Occasionally, a few apoptotic cells were detected in the dorsal aspect of the retina (not shown). This low level pattern of cell death correlates well with previous reports of cell death in E11.5 mouse embryos [40]. Levels of TUNEL-labelled cells were compared in BMP4-treated and contralateral control eyes. No significant differences in apoptosis were detected in the ventral (domain 4-containing) or the central retina between BMP4-treated and control eyes, suggesting that loss of *Vax2 *expression was not associated with apoptosis (Fig. 7D,E). Instead, more TUNEL-positive cells were found at the vitreal edge in the dorsal retina (domain 1; Fig. 7D,E), close to the source of endogenous BMP4 expression and where BMP4 levels appear highest after exogenous BMP4 addition. In total, 79 TUNEL-positive cells were detected in the dorsal region of BMP4-treated retina compared with 26 in contralateral non-treated eyes (n = 26 sections from 5 embryos; TUNEL-positive cells: p = 0.036 by 2-way ANOVA; TUNEL index: p = 0.04 by ANCOVA; [see Additional File 2C]). We also carried out BMP4-bead implantations using higher doses of BMP4 to test whether this increased the level of apoptosis. Widespread apoptosis and tissue ablation were detected throughout the neural retina (Fig. 7G). Therefore, while excess BMP4 can induce widespread apoptosis in the retina, the level of BMP4 that caused ventral shifts of gene expression domains, was only associated with a modest increase in apoptosis in the dorsal eye.

Taken together these data support the view that BMP4 signaling from the dorsal eye primordia is important for defining gene expression domains across the D-V axis of the retina. An increase in the level of BMP4 in the optic cup causes a shift in gene expression domains and expands the dorsal retinal territory at the expense of the ventral territories. These changes in gene expression domains fundamentally alter the molecular character of the optic cup. Furthermore, these data suggest that alterations in the BMP4 signaling gradient disrupt differential regulation of cell proliferation across the D-V axis, thus affecting growth and shape of the optic cup.

## Discussion

Using whole embryo culture and bead implantation, our analyses have provided new evidence for the critical importance of the level of BMP4 signaling for D-V patterning and growth of the developing mouse eye. Our data suggest that BMP4 patterns the optic cup by dosage dependent regulation of target genes, *Tbx2*, *Tbx3 *and *Tbx5*.

### A conserved genetic pathway: BMP4 regulation of Tbx2 subfamily genes

In whole embryo culture experiments, exogenous BMP4 induced *Tbx2, Tbx3 *and *Tbx5 *expression, while Noggin repressed/reduced *Tbx2 *and *Tbx5 *expression in the developing retina, providing strong support for the idea that BMP4 signaling regulates T-box gene expression during mouse eye development. This is the first demonstration that all three members of the Tbx2 subfamily, *Tbx2*, *Tbx3 *and *Tbx5 *genes are downstream targets of BMP signaling in the mouse eye. A recent paper reported loss of *Tbx5 *expression in the optic vesicles of *Bmp4*^-/- ^mice [61]. Previous reports have demonstrated BMP2/4/Dpp regulation of Tbx2 subfamily genes in the heart, limb and retina in other vertebrates and of *omb *in *Drosophila *[41,49,52-54,68]. Considered together, these data indicate that BMP2/4 regulation of Tbx2 subfamily genes is an ancient and evolutionarily conserved pathway, which has been recruited to regulate development and patterning of different tissues in vertebrates and invertebrates.

### In the developing eye, *Tbx2*, *Tbx3 *and *Tbx5 *show differential responses to BMP4 signaling

Our data suggest that in the eye, each T-box gene responds to BMP4 in a different manner. They are consistent with a model in which *Tbx5 *responds to BMP4 close to the dorsal signaling source, while *Tbx2 *and *Tbx3 *can be induced in a wider area by lower levels of BMP4 secreted from the source. Based on the expression pattern of *Bmp4 *in the dorsal region of the optic vesicle and the optic cup, it is likely that BMP4 protein concentrations will be high in the dorsal and low in the ventral region of the eye; indeed the dorsal retina shows high levels of BMP signaling activity as determined by immunohistochemistry for Phospho-Smad 1/5/8 proteins.

The different spatial response that each T-box gene showed and their close evolutionary relationship support a mechanism of direct regulation by BMP4 signaling, although we have not excluded the possibility that other intermediate factors regulate these genes. Significantly, a recent study showed that the *Tbx3 *promoter is directly regulated by BMP Smads, in chromatin immunoprecipitation experiments using extracts from embryonic hindlimbs, as well as cotransfection assays [69]. In *Drosophila*, molecular dissection of *cis*-regulatory sequences of *omb*, the homologue of vertebrate *Tbx2/3*, revealed a Dpp response element [70] suggesting that *omb *may also be a direct target of Dpp signaling. Interestingly, *Tbx3 *responded more vigorously than *Tbx2 *to exogenous BMP4 administered to the lens, extending all the way to the ventral retina, whereas *Tbx2 *did not extend as far and may need other factors or is being inhibited by factors present in the ventral retina. The fact that *Tbx3 *expression is initially more restricted than the *Tbx2 *expression domain, yet after exogenous BMP4 treatment it shows the greatest expansion suggests that other factors are regulating the expression of T-box genes along with BMP4.

It has been proposed that *Tbx5 *represses *cVax *and vice versa in the chick based on overexpression studies [21,41]. The cascade in the mouse appears to differ, as with increased BMP4 levels, we observed repression of *Vax2 *without ventral expression of *Tbx5*. Also, in *Vax2 *null mice, the *Tbx5 *expression domain is unchanged [71]. Moreover the separation of *Vax2 *and *Tbx5 *expression in two non-overlapping domains (domains 1 and 4) indicate that these factors are unlikely to control each other's expression.

### Tbx2 subfamily genes as regulators of apoptosis and proliferation

Downstream targets regulated by the *Tbx2*, *Tbx3 *and *Tbx5 *transcription factors have not been identified in the eye. Several *in vitro *studies implicate these genes in the regulation of cell proliferation and apoptosis. Ectopic expression of *TBX5 *in osteosarcoma cells inhibits cell proliferation and induces apoptosis [72,73]. *TBX2 *gene amplification has been detected in primary human breast cancer tumours, pancreatic cancer cell lines, and its overexpression detected in melanoma cell lines [74-78]. Both *TBX2 *and *TBX3 *repress the expression of p14^ARF^, and there is evidence for the repression of other cyclin dependent kinase inhibitors p21, p16^INK4a^, and p15^INK4b ^by *TBX2 in vitro *[74,79-81]. Moreover, both *TBX2 *and *TBX3 *show anti-senescence properties [74,82], while a dominant negative form of Tbx2 can induce senescence in a melanoma cell line [76].

During embryogenesis, a similar pro-proliferative role has not been identified for these genes. In fact, it has been shown that the expression of p21, p19^ARF^, p16^INK4a^, and p15^INK4b ^is normal in mice with a targeted mutation of *Tbx2*, and there is no evidence for a genetic interaction between *Tbx2 *and p53 (*Trp53*) [83]. Furthermore, *Tbx2 *expression has been associated with a low rate of proliferation in the atrioventricular canal during heart development [84]. *Tbx5 *has also been associated with the regulation of cell proliferation during embryogenesis. Misexpression of *TBX5 *in the chick heart leads to inhibition of cell proliferation [72] and in the limb, ectopic expression of *Tbx5 *(or *Tbx4*) leads to a truncated limb phenotype due to cell proliferation arrest [68].

We observed a dosage sensitive increase in the amount of apoptosis in BMP4-treated eyes, which correlates with *Tbx5 *expression in the dorsal retina, but not with the ectopic *Tbx2 *or *Tbx3 *expression in the ventral retina. Alternatively, the homeobox gene *Msx2*, which is normally expressed in the dorsal neural retina [40,65], could be mediating the apoptotic effect of the elevated BMP4 levels in the dorsal neural retina. Msx genes mediate BMP induced apoptosis during development of other organs such as the hindbrain and the limbs [57,62]. The different response of *Msx2 *and the T-box genes to BMP4 treatment suggests that these genes do not regulate each other. If *Tbx2 *acts to reduce proliferation during optic cup formation, as recently found in the developing heart [84], then *Tbx2 *expansion into the ventral retina may contribute to the observed reduction in levels of proliferation after BMP4 treatment.

### BMP4 dosage in the regulation of eye size and shape

Addition of exogenous BMP4 in the mouse optic cup in embryo cultures resulted in a reduction in retinal volume and alterations in eye shape. In chick and *Xenopus*, malformation of the ventral retina has previously been described after overexpression of BMP4 by electroporation [41,52]. Increases in gene dosage, as well as loss of function are known to cause congenital eye malformations in humans and mice, for example in the case of the transcription factor genes *Pax6 *and *Foxc1 *[85,86]. Our findings suggest that increased levels of BMP4 signaling may be detrimental; in the human population, such changes could occur, for instance, via genetic variation in regulatory sequences, gene duplication, or gain of function mutations. The recent report that anophthalmia occurs in embryos from crosses between *Bmp4 *heterozygous mutants and mice homozygous for a disrupted allele of *Twisted gastrulation *(*Tsg*), a BMP binding protein that is thought to enhance BMP4 signaling in the eye [87], also indicates that excess BMP4 signaling may be as harmful as loss of function.

The adverse consequence of reduced levels of BMP4 signaling for eye development has been explored in different systems. That optic vesicle development is arrested in *Bmp4 *null mice [26] first indicated the importance of *Bmp4 *for normal growth. We observed an expansion of optic stalk and a reduction in the neural retina after addition of the BMP antagonist, Noggin, close to the dorsal BMP4 signaling source in the mouse optic cup. In the chick embryo, misexpression of Noggin induces primarily ventral abnormalities that include optic stalk hyperplasia [42]. Conditional gene inactivation has also been used to analyse the effect of loss of the serine/threonine kinase membrane bound BMP receptors; *BmprIa*, *BmprIb*, and *BmprII *are expressed in the developing mouse eye [26,88,89]. Whereas targeted deletion of *Bmpr1b *alone disrupts the ability of many ventrally located ganglion cells to enter the optic nerve head [88], conditional *Bmpr1a*^-/*fx*^/*Bmpr1b*^-/-^*;cre *double mutant embryos exhibit more profound structural abnormalities due to excess apoptosis and reduced proliferation at E11.5, and show anophthalmia at birth [61]. In these mutants, the loss of Bmpr1a and Bmpr1b likely interfered with BMP4 signaling, as well as signaling by BMP7, BMP2 and BMP3, from non-neural retinal sources [90].

The findings of altered eye size and shape with loss and gain of BMP4 function, suggest that a BMP4 signaling gradient mediates control of proliferation across the D-V axis during optic cup morphogenesis. With exogenous BMP4, we observed a significantly decreased level of retinal cell proliferation in both the dorsal and the ventral retina. Regional differences in proliferation have recently been found during optic cup growth [4,5], and we found that the exogenous BMP4 acted to equalise the differences in levels of proliferation across the D-V axis. Other studies have linked excess BMP4 to reduced proliferation and increased apoptosis. For example, the BMP expressing region of the mouse telencephalon has a lower proliferative activity and higher level of apoptosis than the rest of the forebrain, and BMP4-soaked beads can induce apoptosis in telencephalic explants [66]. In the chick, exogenous BMP4 applied to the dorsal optic cup, or at early stages to the telencephalon also increases apoptosis [40,91]. However, in the same studies, addition of BMP4 to chick retinal cultures at later stages induced proliferation [40], while reduced levels of BMP signaling (via *Noggin *misexpression) in the telencephalon was associated only with a decrease in proliferating cells [91], highlighting that the cellular response to BMP4 signaling appears stage and context dependent. In our study, only at higher doses of BMP4 was widespread apoptosis induced.

### Threshold-specific requirements for BMP4 in optic cup development

Our data show that a major role for dorsal BMP4 signaling in the developing mouse optic cup is to regulate gene expression across the D-V axis. It suggests that BMP4 may be critically important for providing a signaling gradient, which is used to demarcate gene expression domains across the D-V axis, with retinal cells expressing different transcription factor genes in response to different threshold levels of BMP4 activity. Variations in Smad binding site distribution between target genes or levels of other active cofactors are likely to contribute to the differential gene responses.

Exogenous BMP4 treatment prior to optic cup formation affected *Tbx2 *and *Tbx5*, but not *Vax2 *or *Tbx3*, and T-box gene expression changes were not induced in the ventral retina in contrast to the effect of exogenous BMP4 addition to the lens. These differences may be due to the different developmental stage, and/or the different range of BMP signaling from bead implantation in the lens compared with the extraocular mesenchyme. In the latter case the different intervening tissue between the bead and the retina that is exposed to the BMP signal may be significant; when a bead is implanted in the lens the intervening tissue is the lens, whereas from a bead located in the mesenchyme, the BMP signal has to pass through the presumptive RPE. We were only able to detect increased levels of Phospho-Smad 1/5/8 immuno-labeling after BMP4 bead implantation in the lens, which correlates with our findings that lens bead implantation showed the most dramatic effect on gene expression domains. In this study it was not possible to distinguish if the BMP gradient regulates the expression of the Tbx2 subfamily genes directly or indirectly. Rather than the BMP gradient being directly responsible for the nested expression domains it is possible that there are other secondary molecules induced by BMP that carry the signal. However, our data considered together with recent data showing that the *Tbx3 *promoter is directly regulated by Smads [69] strongly support the idea that these genes respond directly to a BMP4 signaling gradient.

Although there are some differences in the specific gene expression patterns between chick and mouse, the broadly conserved pattern of gene expression domains across the D-V axis of the optic cup suggests that compartment-like units of clonally related cells defined by restricted gene expression domains, as previously described in the chick [6], may also exist in the mouse. If this is the case, then our data suggests that altering the BMP4 concentration in the eye re-specifies the compartments of the retina along the D-V axis; increased BMP causes compartments to express markers normally expressed by their more dorsal neighbours, although the response to BMP4 is not uniform in the different genes examined. Our analysis of proliferation and apoptosis support the conclusion that these changes in gene expression domains reflect changes in the identity of retinal territories rather than tissue loss. Instead of being made up of domains 1, 2, 3 and 4, the post BMP-treatment eye consists only of domains 1, 2 and a modified domain 3 (domain 3* that lacks *Tbx2 *and *Vax2 *expression but expresses *Tbx3*). The importance of correct spatial restriction of gene expression for topographic mapping of retinal ganglion cells to the brain is well established [21,41,92]; our data suggests it is also important in regulating regional growth of the developing optic cup.

Our finding that exogenous BMP4 not only extends expression of the dorsally expressed T-box genes in the optic cup, but also reduces the ventral marker *Vax2 *support the idea that BMP4 signaling has a long-range action across the D-V axis, and that BMP4 may repress *Vax2 *expression. BMP inhibitors, in particular *Ventroptin *and *Dan*, are known to be expressed in a ventral-high gradient in the chick eye [93,94], although these expression patterns have not been reported in the mouse. The role of such inhibitors may normally be to block long range BMP4 signaling from acting in the ventral-most region (domain 4), allowing *Vax2 *expression, and preventing induction of dorsally expressed genes. In our study, increased levels of BMP4 at optic cup stage are apparently sufficient to overcome endogenous inhibitors, leading to *Vax2 *repression and induction of T-box gene expression in the ventral retina.

Also consistent with the proposal that BMP regulates *Vax2*, the recent informative study by Murali et al [61], showed that with reduced levels of BMP signaling, *Tbx5 *expression was lost whereas *Vax2 *expression was expanded into the dorsal mouse retina in *Bmpr1a*^-/*fx*^*/Bmpr1b*^+/-^*;Cre *double mutants. In chick, retroviral overexpression of *Bmp4 *was previously shown to repress expression of *Ventroptin *[94]. Alternative to direct regulation of *Vax2 *by BMP4, interaction with other ventral signaling pathways could be important, as in zebrafish and chick, hedgehog signaling from the ventral midline contributes to regulation of the *Vax *gene [95,96]. Mouse embryos cannot be cultured *ex utero *for long enough to analyse the effect of the shifted gene expression domains on late stages of eye development. Nevertheless, the effect of loss of *Vax2 *expression in the ventral eye has already been carefully studied in *Vax2 *null mice and shown to cause profound defects in ventral eye formation and coloboma [20,22,97].

## Conclusion

Our data suggest that BMP4 signaling from a dorsal site of expression, patterns the mammalian optic cup across the entire D-V axis, by dosage dependent regulation of target genes, *Tbx2*, *Tbx3*, *Tbx5 *and *Vax2*. Our findings fit with the idea that a BMP4 signaling gradient across the developing optic cup is interpreted to create domains of BMP4 target gene expression. A conserved genetic pathway is emerging whereby BMP4 regulates members of the Tbx2 subfamily in different tissues and organisms during organogenesis. Increasing evidence suggests that *Tbx2 *and *Tbx3 *may regulate proliferation in cancer and in development and our data is consistent with a role for these T-box genes downstream of BMP4 in regulating proliferation of the optic cup. Upon increasing the level of BMP4 signaling, growth of the embryonic eye was perturbed resulting in a reduction in retinal volume as well as the D-V and N-T axial lengths of the optic cup. That precise levels of BMP4 signaling are critical for normal mammalian eye development is becoming progressively more clear; it is likely that altered dosage of BMP4 is a mechanism underlying human congenital eye defects.

## Methods

### Whole mouse embryo culture

Mouse embryos at E9.5, E10.5, and E11.5 were obtained from timed matings of CBA/Ca mice. Days on which plugs were found after overnight matings were considered to be E0.5. Embryos were dissected and cultured based on the method of D.L Cockroft [58,98]. E9.5 embryos were cultured with intact yolk sacs, whereas E10.5 and E11.5 embryos were cultured outside the yolk sac. For E10.5 and E11.5 embryos, a small incision was made in the yolk sac adjacent to the placenta, avoiding large blood vessels. The yolk sac and the amnion were then drawn over the head of the embryo. Embryos were incubated at 37°C in a rolling incubator (B.T.C. Engineering, Cambridge, UK) in 3 ml culture medium consisting of 100% rat serum (prepared as described in [98]) for E9.5 embryos or 25% rat serum 75% culture saline (0.12 M NaCl, 4 M KCl, 0.4 mM MgSO_4_, 0.25 mM MgCl_2_, 0.60 mM NaH_2_PO_4_, 1.8 mM CaCl_2_, 11.1 mM glucose, 24 mM NaHCO_3_) for E10.5–11.5 embryos. The medium was manually gassed prior to culture for one minute with a 20% O_2_/5% CO_2 _source for E9.5, and 95% O_2_/5% CO_2 _(Crysoservice Ltd, UK) for E10.5–E11.5. Embryos were cultured for 1 hour before bead implantation.

### Bead implantation

10 μl of Affi-Gel Blue beads, diameter 80–150 μm (Bio-Rad Laboratories, CA, USA), were washed three times in embryo water (Sigma, UK), then incubated in 5 μl of either 100 μgml^-1 ^human recombinant BMP4 (R&D Systems, UK), or 1 mgml^-1 ^Noggin (R&D Systems, UK), or 1 mgml^-1 ^bovine serum albumen (BSA; Promega, USA), for 1 hour at 37°C prior to implantation. High concentration BMP4 beads were prepared as previously described [99]. E9.5 mouse embryos with intact yolk sacs were transferred to a dish of explanting saline (same as culture saline except 0.83 mM glucose, 0.6 mM NaHCO_3_). To enable bead implantation, a small tear was made using watchmaker's forceps (Raymond Lamb, UK) in the yolk sac adjacent to the ectoplacental cone, then an arc was cut using iridectomy scissors around one third of the cone (adjacent to the embryonic head). Watchmaker's forceps were used to tear the amniotic sac and expose the eye region. E10.5 and E11.5 embryos with their yolk sac removed, were rested on their side in explanting saline to expose the eye. The site of bead implantation was pierced using a tungsten needle and then using a mouth-operated glass capillary pipette, a bead was inserted into the extra-ocular mesenchyme (at E9.5 and E10.5) or in the lens vesicle (at E11.5). Following bead implantation, embryos were gassed again for 1 minute and cultured for 18 hours at 37°C. Embryos were scored after culture for blood circulation, number of somites and head length [98]. Embryos were only analysed when circulation was maintained and somite number had increased by at least 6 pairs during culture. Overnight *ex utero *growth of optic vesicle stage embryos (somite stage 24–26) to somite stages 33–35 resulted in optic vesicle invagination. Overnight *ex utero *growth of optic cup stage embryos (somite stage 33–35) to somite stages ≥ 44 resulted in normal growth and pigmentation of the optic cup. For each treatment, viable post culture embryos from at least two litters were analysed in independent experiments. After culture, embryos were fixed overnight in 4% para-formaldehyde (PFA) for analysis.

### Wholemount *in situ *hybridisation

RNA *in situ *hybridisation was performed on 4% PFA fixed cultured, operated and control embryos using standard protocols [100]. For detection of single genes, digoxigenin (DIG)-labelled RNA probes were prepared for *Tbx2*, *Tbx3*, *Tbx5*, *Msx2*, and *Vax2 *using a DIG RNA labeling kit (Roche, Germany) according to the manufacturer's description. For detection of *Tbx2 *and *Vax2 *by double *in situ *hybridisation, *Tbx2 *DIG-labelled and *Vax2 *fluorescein-labelled probes were prepared and hybridised simultaneously. Plasmids used were a 2.2 kb *Tbx2 *cDNA in pBK-CMV, 3 kb *Tbx5 *cDNA in pBK-CMV [50], 1 kb *Tbx3 *cDNA in pB(II)KS (kindly provided by V. Papaioannou, Columbia University), a 700 bp *Vax2 *cDNA in pBS SK (kindly provided by C. Cepko, Harvard Medical School), and a 1000 bp *Msx2 *cDNA in pCR2.1 (kindly provided by P. Sharpe, King's College London). Hybridisation of labelled probe was visualised using alkaline phosphatase conjugated anti-DIG antibody and the colour reaction was developed with NBT/BCIP (Roche, Germany); treated and control eyes of the same embryo were compared ensuring identical reaction conditions. For double *in situ *hybridisation, the *Vax2 *probe was detected with alkaline phosphatase conjugated anti-fluorescein antibody and the colour reaction developed with NBT/BCIP. Embryos were then incubated at 65°C for 30 minutes in TBST (137 mM NaCl, 2.7 mM KCl, 0.25 M Tris.HCl pH 7.5, 1% Tween-20, 2 mM Levamisol), blocked in TBST containing 10% heat-inactivated sheep serum for 1 hour at room temperature and incubated with anti-DIG antibody for detection of the *Tbx2 *probe with INT/BCIP (Roche, Germany).

### Microscopy

Images of whole mount embryos were captured with a Leica MZ FLIII microscope fitted with a Leica DC500 camera using the Leica IM1000 Image manager V1.20 software (Leica, Germany), and imported into Adobe Photoshop (6.0). Vibratome sections (50 μm) prepared after whole mount *in situ *hybridisation were imaged using an Axiophot 2 (Zeiss, Germany) with differential interference contrast (DIC) optics, a Leica digital MZIII camera and Openlab 4.0.4 software (Improvision Ltd., UK). Fluorescent images of sections after immunohistochemistry were digitally captured using an Axiophot (Zeiss, Germany) microscope, ProgRes Cl4 camera (Jenoptik Jena, Germany) and Openlab 4.0.4 software.

### Morphometric analysis

To estimate retinal volume, post-culture embryos were embedded in 0.45% w/v gelatine, 28% w/v egg albumin, 18 % w/v sucrose, 0.2% glutaraldehyde (all from Sigma, UK) in phosphate buffered saline (PBS). Serial transverse vibratome sections (50 μm) of each eye were mounted on slides with 50% glycerol/PBS and glass coverslips. Retinal surface area per section was estimated using Openlab 4.0.4 software. Retinal volume per section was calculated as retinal surface area multiplied by section thickness, and total retinal volume was calculated by addition of retinal volumes of all sections per eye. BMP4-treated eyes were compared to contralateral BSA-treated eyes using the paired t-test. To determine the proximo-distal (P-Di) axis, the distance from the ventricular edge of the retina to the surface ectoderm was measured using Openlab 4.0.4 software on serial sections and the longest distance for each optic cup was compared with the paired t-test. To determine the dorso-ventral (D-V) and naso-temporal (N-T) axial lengths of optic cups after culture, digital images of optic cups captured using identical conditions, were measured using the Photoshop (6.0) measure tool. Operated eyes were compared to contralateral eyes in each embryo using the paired t-test.

### Immunohistochemistry, analysis of cell proliferation, apoptosis, and histology

Phospho-Smad 1/5/8 immunohistochemistry was performed on coronal wax sections of 4% PFA fixed post-culture embryos. Sections (7 μm) were de-parafinised and un-masked by steaming in Declear (Cellmarque, USA) for 40 minutes and then incubated in 3% H_2_O_2 _in TBS (137 mM NaCl, 2.7 mM KCl, 0.25 M Tris pH7.5) containing 0.1% Triton-X100 (BDH, UK) for 10 minutes. Sections were incubated in blocking solution (5% v/v goat serum, 0.15% w/v glycine, 2 mg/ml BSA in TBS-0.1% Triton-X100) for 30 minutes and then in blocking solution containing a 1:100 dilution of the Phospho-Smad1/5/8 antibody (Cell Signaling technology, USA) for 1 hour at room temperature. This was followed by incubation in a 1:250 dilution of Biotinylated goat anti-rabbit (Dako, Denmark) secondary antibody in blocking solution for 1 hour at room temperature. The secondary antibody was detected with the Vectastain ABC kit (Vector Laboratories, USA) and the diaminobenzidine tetrahydrochloride (DAB; Sigma, UK) chromogen according to the manufacturers' descriptions. Sections were counterstained with 0.5% w/v methyl green (BDH, UK). Histology was examined on 7 μm thick coronal wax sections stained with Haematoxylin and Eosin (BDH, UK). Frozen coronal sections of 4% PFA fixed, OCT (BDH, UK) embedded, post-culture embryos were prepared for analysis of cell proliferation and apoptosis. Anti-phospho-histone H3 (pH3) immunohistochemistry was used to detect mitotic cells in the neural retina. Sections were incubated in blocking solution (10% foetal calf serum, 1% bovine serum albumin, 0.1% Tween-20 in PBS) for 1 hour, followed by incubation with primary antibody, rabbit anti-pH3 (1:100 dilution in blocking solution, Upstate, USA), overnight at 4°C. Sections were incubated with FITC-conjugated goat anti-rabbit secondary antibody (1:100 in blocking solution, Jackson Immunoresearch laboratories, USA) and propidium iodide (1 μgml^-1^, Sigma, UK), for 1 hour at room temperature. pH3-positive cells were counted in 3–5 midline sections per eye per embryo (n = 4 embryos). A 2-way Analysis of Variance (ANOVA) was used to test whether the number of pH3 cells per section differed between paired BMP4-treated and contralateral non-treated eyes. The Shapiro-Wilk's test of normality was used to confirm that residual values (observed value – value predicted by the model) were normally distributed. Total cell number per section was counted on digital images in Photoshop (6.0). The effect of total cell number on the number of pH3-positive cells was tested by incorporating total cell number per section as a covariate in the Analysis of Co-Variance (ANCOVA) test. To investigate whether the reduction in the number of mitotic cells is regional along the D-V axis of the neural retina, retinal sections were divided into a dorsal region above the optic stalk (including domains 1, 2 and 3) and a ventral region below the optic stalk (containing the *Vax2 *expression domain 4) and pH3 labelled cells were counted in each region.

TUNEL (Terminal deoxynucleotidyl transferase-mediated dUTP Nick End Labeling, Roche, UK) was used to detect apoptotic cells according to the manufacturer's description. Briefly, frozen sections were permeabilised with 0.1% Triton X-100 in PBS for 2 minutes and incubated at 37 C° for 1 hour with TUNEL mix. Afterwards, sections were counterstained with propidium iodide (1 μgml^-1^, Sigma, UK) in PBS and mounted with CITIFLUOR. The number of TUNEL-positive cells was counted in dorsal, central, and ventral retinal regions in 2–3 midline sections of eyes in five embryos and compared between the BMP4-treated and non-treated contralateral eyes using a 2-way ANOVA; retinal area was incorporated into an ANCOVA to test for the effect of retinal size on the number of TUNEL positive nuclei (TUNEL index).

## Authors' contributions

HB and JKLH carried out the bead implantation and whole embryo culture experiments. HB carried out the proliferation and apoptosis study, the morphometric and statistical analyses, prepared the figures, and assisted with the preparation of the manuscript. JCS conceived of the study and wrote the manuscript. All authors read and approved the final manuscript.

## Supplementary Material

Additional File 1 ***Tbx3*, *Vax2*, *Msx2 *expression and Phospho-Smad 1/5/8 localisation after alteration of BMP4 signaling in embryo culture by addition of exogenous Noggin or BMP4. (A, B) **Post-culture embryo showing normal dorsal *Tbx3 *expression in the control non-treated and Noggin-treated optic cups respectively. **(C, D) **Post-culture embryo showing normal ventral *Vax2 *expression in the control non-treated and Noggin-treated optic cups respectively. **(E) **The boundary of *Bmp4 *expression (arrow) in the dorsal neural retina of an E11.5 embryo. **(F) **Post-culture embryo showing high levels of Phospho-Smad 1/5/8 labeling in the dorsal neural retina and in the lens. The extent of the highest level of BMP signaling in the retina is demarcated by arrow. **(G) **BMP4-treated contralateral eye showing a wider region of high level Phospho-Smad 1/5/8 labeling (arrow). **(H, I) **Another example of the extension of BMP signaling in the BMP4-treated optic cup (I) as compared with the control eye (H) in a post-culture embryo. Sections H and I are counterstained with methyl green. **(J) **Post-culture embryo showing normal *Msx2 *expression restricted to the dorsal neural retina and lens. **(K) **BMP4-treated contralateral eye showing widespread induction of *Msx2 *expression in the lens and the optic cup. Noggin or BMP4-soaked beads are indicated with asterisks (in A-K) and domains of gene expression are demarcated with arrows (in A-D). E-I show coronal sections of eyes. Abbreviations: L, lens vesicle; NR, neural retina. Click here for file

Additional File 2 **Analysis of pH3 and TUNEL in the dorsal and ventral retina after BMP4 treatment. (A, B) **Graphs show the number of mitotic cells per section per eye in the dorsal (A) and ventral (B) regions of BMP4-treated optic cups compared with contralateral control optic cups (n = 44 sections from 4 embryos; p = 0.007 in dorsal, p = 0.018 in ventral by ANOVA). **(C) **Graph shows the programmed cell death index (per section per eye) in the dorsal neural retina of BMP4-treated and contralateral control eyes (n = 26 sections in 5 embryos; p = 0.044 by ANCOVA). Data was Ln transformed for normalisation. Click here for file
