# Supplementary figures and images for: The level of BMP4 signaling is critical for the regulation of distinct T-box gene expression domains and growth along the dorso-ventral axis of the optic cup
Source: BMC Dev Biol. 2006 Dec 15;6:62. doi: 10.1186/1471-213X-6-62 (PMC1764729; doi:10.1186/1471-213X-6-62)

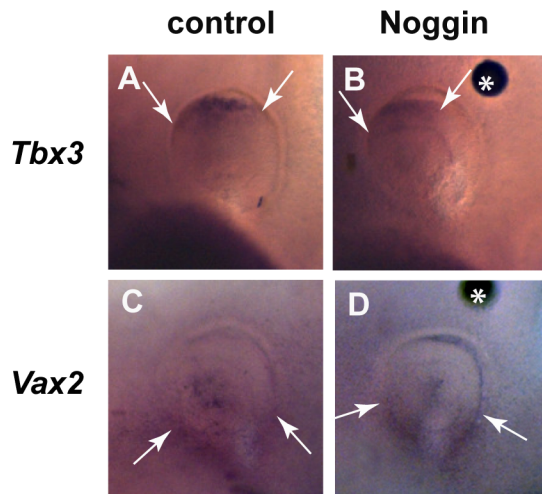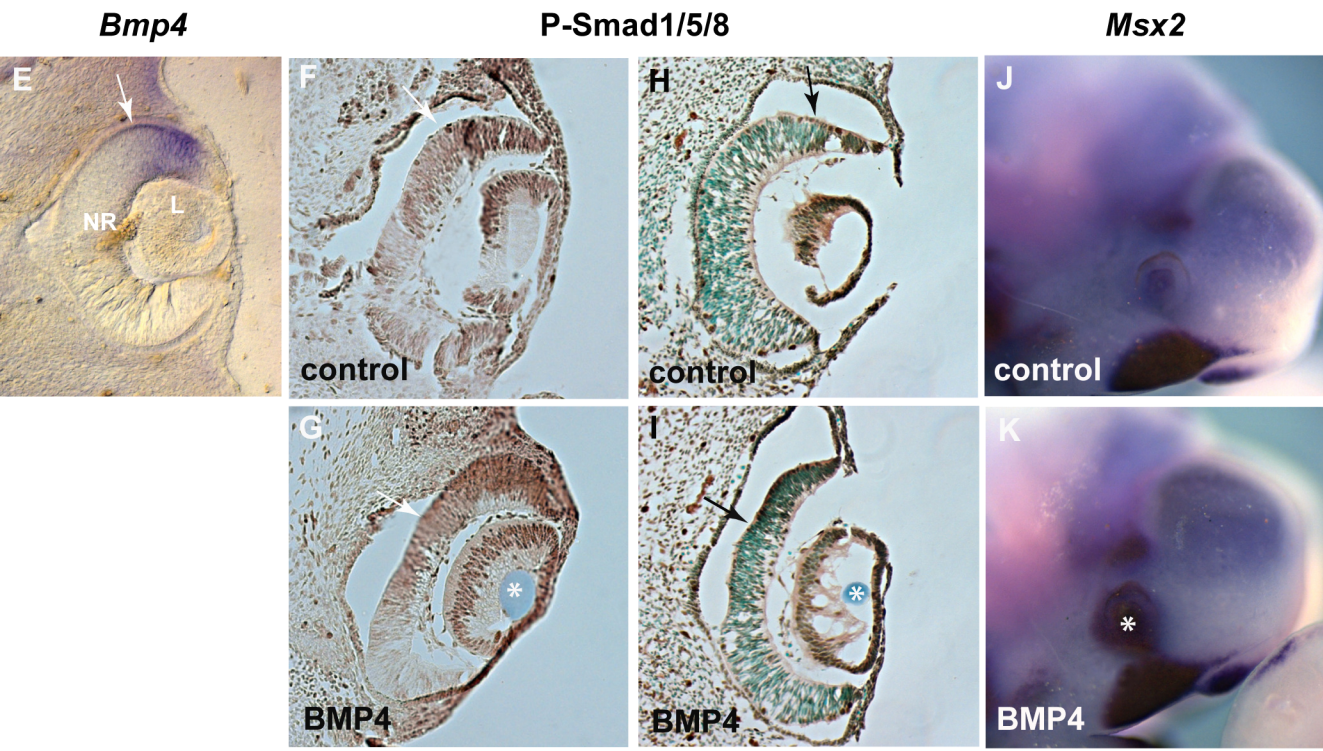

Supplement: Additional File 1 — Tbx3, Vax2, Msx2 expression and Phospho-Smad 1/5/8 localisation after alteration of BMP4 signaling in embryo culture by addition of exogenous Noggin or BMP4. (A, B) Post-culture embryo showing normal dorsal Tbx3 expression in the control non-treated and Noggin-treated optic cups respectively. (C, D) Post-culture embryo showing normal ventral Vax2 expression in the control non-treated and Noggin-treated optic cups respectively. (E) The boundary of Bmp4 expression (arrow) in the dorsal neural retina of an E11.5 embryo. (F) Post-culture embryo showing high levels of Phospho-Smad 1/5/8 labeling in the dorsal neural retina and in the lens. The extent of the highest level of BMP signaling in the retina is demarcated by arrow. (G) BMP4-treated contralateral eye showing a wider region of high level Phospho-Smad 1/5/8 labeling (arrow). (H, I) Another example of the extension of BMP signaling in the BMP4-treated optic cup (I) as compared with the control eye (H) in a post-culture embryo. Sections H and I are counterstained with methyl green. (J) Post-culture embryo showing normal Msx2 expression restricted to the dorsal neural retina and lens. (K) BMP4-treated contralateral eye showing widespread induction of Msx2 expression in the lens and the optic cup. Noggin or BMP4-soaked beads are indicated with asterisks (in A-K) and domains of gene expression are demarcated with arrows (in A-D). E-I show coronal sections of eyes. Abbreviations: L, lens vesicle; NR, neural retina. [file 1471-213X-6-62-S1.pdf]

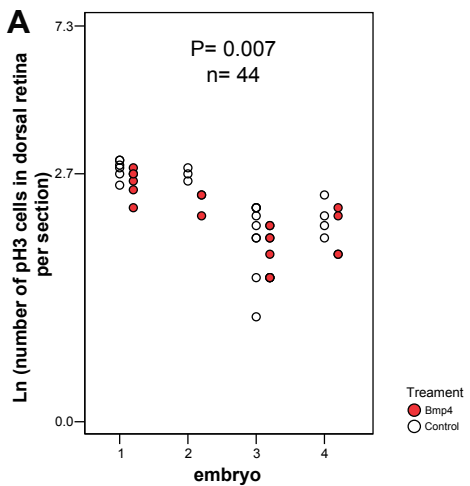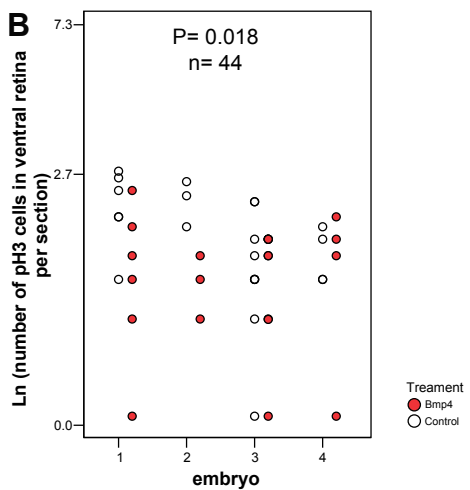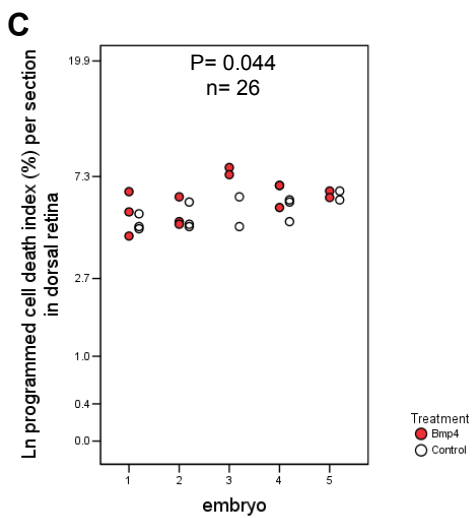

Supplement: Additional File 2 — Analysis of pH3 and TUNEL in the dorsal and ventral retina after BMP4 treatment. (A, B) Graphs show the number of mitotic cells per section per eye in the dorsal (A) and ventral (B) regions of BMP4-treated optic cups compared with contralateral control optic cups (n = 44 sections from 4 embryos; p = 0.007 in dorsal, p = 0.018 in ventral by ANOVA). (C) Graph shows the programmed cell death index (per section per eye) in the dorsal neural retina of BMP4-treated and contralateral control eyes (n = 26 sections in 5 embryos; p = 0.044 by ANCOVA). Data was Ln transformed for normalisation. [file 1471-213X-6-62-S2.pdf]
